# Molecular Mode of Action and Role of TP53 in the Sensitivity to the Novel Epothilone Sagopilone (ZK-EPO) in A549 Non-Small Cell Lung Cancer Cells

**DOI:** 10.1371/journal.pone.0019273

**Published:** 2011-04-29

**Authors:** Sebastian Winsel, Anette Sommer, Julia Eschenbrenner, Kevin Mittelstaedt, Ulrich Klar, Stefanie Hammer, Jens Hoffmann

**Affiliations:** 1 Global Drug Discovery, Bayer Healthcare, Berlin, Germany; 2 Institute for Chemistry-Biochemistry, Freie Universität Berlin, Berlin, Germany; 3 Medical Biotechnology, VTT Technical Research Centre of Finland, Turku, Finland; 4 Institut für Biotechnologie, Technische Universität Berlin, Berlin, Germany; 5 Department of Medicine, The University of Melbourne, Melbourne, Australia; 6 Experimental Pharmacology, Max-Delbrueck-Center for Molecular Medicine, Berlin, Germany; Vanderbilt University Medical Center, United States of America

## Abstract

Sagopilone, an optimized fully synthetic epothilone, is a microtubule-stabilizing compound that has shown high *in vitro* and *in vivo* activity against a broad range of human tumor models. We analyzed the differential mechanism of action of sagopilone in non-small cell lung cancer cell lines *in vitro*. Sagopilone inhibited proliferation of non-small cell lung cancer cell lines at lower nanomolar concentration. The treatment with sagopilone caused strong disturbances of cellular cytoskeletal organization. Two concentration-dependent phenotypes were observed. At 2.5 nM sagopilone or 4 nM paclitaxel an aneuploid phenotype occur whereas a mitotic arrest phenotype was induced by 40 nM sagopilone or paclitaxel. Interestingly, treatment with 2.5 nM of sagopilone effectively inhibited cell proliferation, but - compared to high concentrations (40 nM) - only marginally induced apoptosis. Treatment with a high versus a low concentration of sagopilone or paclitaxel regulates a non-overlapping set of genes, indicating that both phenotypes substantially differ from each other. Genes involved in G2/M phase transition and the spindle assembly checkpoint, like Cyclin B1 and BUBR1 were upregulated by treatment with 40 nM sagopilone. Unexpectedly, also genes involved in DNA damage response were upregulated under that treatment. In contrast, treatment of A549 cells with a low concentration of sagopilone revealed an upregulation of direct transcriptional target genes of TP53, like CDKN1A, MDM2, GADD45A, FAS. Knockdown of TP53, which inhibited the transcriptional induction of TP53 target genes, led to a significant increase in apoptosis induction in A549 cells when treated with a low concentration of sagopilone. The results indicate that activation of TP53 and its downstream effectors like CDKN1A by low concentrations of sagopilone is responsible for the relative apoptosis resistance of A549 cells and might represent a mechanism of resistance to sagopilone.

## Introduction

Lung cancer is one of the leading causes of cancer death worldwide, as it is often only diagnosed at advanced stages and displays a high degree of resistance to the chemotherapeutic regimens used. [1]–[5]. Sagopilone (SAG) is a fully synthetic epothilone, currently in clinical development that was optimized to overcome limitations frequently associated with taxanes, conventional tubulin-binding agents (TBAs) as for example MDR mediated resistant mechanisms [6]. SAG has demonstrated high *in vitro* and *in vivo* activity in a range of tumor models compared with paclitaxel (PAC) and other commonly used chemotherapeutic agents [6]. With strong anti-tumor activity been observed in NSCLC (non-small cell lung cancer) cell lines *in vitro* and primary human NSCLC mouse xenograft models, SAG may provide a potential new treatment opportunity for NSCLC [7].

Several studies describe predictive markers of response for NSCLC cell lines, as expression of the excision repair cross-complementation group 1 (ERCC1) gene for platinum compounds [8] or the epidermal growth factor receptor (EGFR) mutational status for the EGFR tyrosine-kinase inhibitors (gefitinib and erlotinib) [9]; [10]. With regard to resistance to TBAs, reports on cell lines selected for PAC resistance through long-term culture in the presence of the drug showed increased levels of TUBB3 (beta III tubulin) protein expression [11]. In contrast, their patupilone (epothilone B) resistant counterpart, incubated in the same manner, showed low TUBB3 protein expression [11]. These data suggest that TUBB3 contributes to the cellular resistance towards PAC but not to epothilone. As a consequence there is a need for other markers that can predict SAG response.

Mutations in tumor-suppressor genes, which plays a central role in cellular response to DNA damage, cell cycle regulation, and apoptosis [12], were found in about 50% of all NSCLC cases [13]. However, the role of TP53 in response to TBAs like PAC has been contested: Some groups reported no correlation between TP53 mutational status and sensitivity to PAC [14]; [15], while others observed that lack of TP53 activity resulted in increased chemosensitivity to PAC [16]; [17]. These findings suggest that the TP53 mutational status and altered TP53 activity might influence the sensitivity of cells to SAG. To address this question, we have analyzed the influence of TP53 on the ability of SAG to induce apoptosis in an NSCLC model *in vitro*.

The identification of stratification biomakers or drug- or drug target-related response markers might considerably improve the outcome of the therapy and would lead to a shift towards more tailored therapies against specific disease types [18]. The optimal treatment for patients suffering from NSCLC will increasingly rely on biomarker analysis to identify the patient population who will benefit most from a certain mono- or combination therapy. Nevertheless, biomarker identification and validation remains a major challenge [19] and it is therefore important to accompany the development of new therapeutic agents for patients with NSCLC with research on patient stratification and the identification and validation of clinical biomarkers which predict response. As part of this process, it is vital to understand how the activity of promising new agents is influenced by alterations in key cellular pathways, and vice versa.

The aim of our translational program was to examine the activity of SAG *in vitro* in a panel of NSCLC cell lines, to further analyze its mechanism of action and to compare it with the effects of PAC and to investigate possible resistance mechanisms as well as predictors of response based on gene expression profiling.

## Materials and Methods

### Cells and compounds

Human lung carcinoma cell lines (A549, NCI-H1437, NCI-H23, NCI-H522, NCI-H226, NCI-H460) were obtained from the American Type Culture Collection (ATCC) and cultured according to recommended protocols. SAG was synthesized at Bayer Healthcare Laboratories through total syntheses. PAC was purchased from Sigma-Aldrich (Munich, Germany). The pan-caspase inhibitor ZVAD.fmk was purchased from Bachem, (Heidelberg, Germany). All media and supplements for cell culture were purchased from Biochrom AG (Berlin, Germany). Stock solutions were prepared as previously described [20]. For selection of stably transduced cell lines Hygromycin was purchased from Roche (Mannheim, Germany).

### Tumor cell proliferation assay

The effect of SAG or PAC on the proliferation of lung cancer was assessed using a cell proliferation assay based on staining cells with crystal violet as described before [20]. IC50 values were calculated from three independent experiments using the Sigmaplot software (SPSS, Friedrichsdorf, Germany).

### *In vitro* analysis of effects on the cytoskeleton, the cell cycle and apoptosis

A549 cells were incubated with vehicle (ethanol 0.1%), 2.5 nM or 40 nM SAG for 20 h, fixed with 4% paraformaldehyde and stained with a monoclonal mouse anti-α-tubulin antibody (1∶1000) (Sigma-Aldrich), Alexa Fluor® 488-linked goat anti-mouse IgG secondary antibody (1∶250) (Invitrogen Inc., Carlsbad, CA, USA) and DRAQ5 (Biostatus, Leicestershire, UK), according to standard protocols. Fixed and stained cells were analyzed using a Zeiss LSM 510 META microscope (Carl Zeiss AG, Jena, Germany) equipped with a Plan-Apochromat® 63x/1.4 (oil DIC) objective. Zeiss LSM software (version 3.0 SP3) was employed for confocal imaging.

Fluorescence-activated cell sorter (FACS) analysis was performed to determine cell cycle distribution of SAG- or PAC-treated cells. Cells were incubated with SAG at the indicated concentrations or vehicle for 18 h, fixed with 70% ethanol, and stained with 50 µg/mL propidium iodide (PI) (Sigma-Aldrich). Cellular DNA content was determined by flow cytometry using the BD FACSCalibur™ (Becton, Dickinson and Company, San Jose, CA, USA) and data were analyzed with the CellQuest™ software (Becton, Dickinson and Company). To investigate apoptosis by FACS, A549 cells were incubated continuously for 72 hrs with the indicated concentrations of SAG or vehicle, trypsinized and stained with DiOC_6_(3) (3,3′-dihexyloxacarbocyanine iodide) (Invitrogen Inc.) and PI as described before [21].

### Quantitative Real-Time PCR and Western Blot

RNA was extracted using RNeasy Mini Kit (Qiagen, Hilden, Germany), cDNA was generated using SuperScript First Strand Synthesis System (Invitrogen Inc.). Real-time PCR was performed with gene expression assays from Applied Biosystems: p21 (#Hs00355782_m1), TP53 (#Hs00153340_m1), Cyclin B1 (#Hs00259126_m1), BUBR1 (Hs00176169_m1), FAS (Hs00163653_m1), GADD45A (Hs00169255_m1), MDM2 (Hs00242813_m1), and HPRT (#4326321E) as endogenous control. Reactions were set up in triplicates using the TaqMan FAST Universal PCR Mastermix and recorded in a 7500 Fast Real-Time PCR-System (Applied Biosystems). The relative expression of each gene was quantified according to the comparative threshold cycle method (ΔΔ _Ct_ method) with equal amplification efficiencies of the target and the endogenous control.

Proteins were extracted using M-PER Mammalian Protein Extraction Reagent (Pierce, Perbio Science, Bonn, Germany). The protein concentrations of the lysates were determined with the BCA Protein Assay Kit (Pierce) according to the manufacturer's instructions. Equal amounts of proteins were separated on a 4–12% Bis-Tris gel (Invitrogen) in an XCell SureLock electrophoresis chamber (Invitrogen) filled with MOPS SDS running buffer and transferred to a PVDF membrane (Invitrogen) according to the manufacturer's instructions. After blocking of unspecific binding sites, the membrane was probed with antibodies specific for CDKN1A (abcam, #16767), TP53 (BD Pharmingen, #15801A), BUB1B (BD Transduction Laboratories, # 612502), Cyclin B1 (BD Pharmingen, # 554176), γH2AX (upstate, # 05-636), PARP (BD Pharmingen, # 551024), phospho-Ser/Thr-MPM-2 (upstate # 05-368), and GAPDH (Advanced ImmunoChemical, # RGM2/Clone 6C5) as loading control.

### RNA extraction for gene expression analysis

A549 cells were seeded in 10 cm cell culture plates and allowed to attach overnight. The cells were then treated with medium containing either 2.5 nM SAG, 40 nM SAG, 4 nM PAC or 40 nM PAC, respectively, vehicle (ethanol 0.1%), or were left untreated for 18 hours. Total RNA was extracted using the RNeasy Mini Kit (Qiagen, Hilden, Germany) including a DNase I (Qiagen) step to eliminate genomic DNA. Total RNA was checked for integrity using the RNA LabChips on the Agilent Bioanalyzer 2100 (Agilent Technologies Inc., Palo Alto, CA, USA) and the concentration was determined on a Nanodrop spectrophotometer (Peqlab, Erlangen, Germany). All RNA samples had high RNA integrity numbers [**22**] larger than 9.5.

### Affymetrix GeneChip® analysis

The One-Cycle Eukaryotic Target Labeling Kit (Affymetrix Inc., Santa Clara, CA, USA) was used according to the manufacturer's instructions. Briefly, 2 µg of high quality total RNA was reverse-transcribed using a T7 tagged oligo-dT primer for the first-strand cDNA synthesis reaction. After RNase H-mediated second-strand cDNA synthesis, the double-stranded cDNA was purified and served as template for the subsequent *in vitro* transcription reaction which generates biotin-labeled complementary RNA (cRNA). The biotinylated cRNA was then cleaned up, fragmented and hybridized to GeneChip HGU133Plus2.0 expression arrays (Affymetrix, Inc., Santa Clara, CA, USA.), which contain 54675 probe sets. The GeneChips were washed and stained with streptavidin-phycoerythrin on a GeneChip Fluidics Station 450 (Affymetrix). After washing, the arrays were scanned on an Affymetrix GeneChip 3000 scanner with autoloader. A total of 30 HGU133Plus2.0 arrays were processed with n = 5 biological replicates for all treatment groups.

Expression analyses were performed with the Expressionist Pro 4.0 software (Genedata AG, Basel, Switzerland). The quality of the data files (CEL format) containing probe level expression data was checked and refined using the Expressionist Refiner software (Genedata AG). The refiner process was performed by clustering of samples on feature intensity level. This allows the identification of possible outliers on feature intensity level. Subsequently, refined CEL files were condensed with MAS5.0 algorithm (Affymetrix) and LOWESS normalized using all experiments as a reference. The normalized expression data sets were loaded into the CoBi database (Genedata) and analyzed with the Genedata Expressionist software. Principle Component Analysis (PCA) and hierarchical clustering was performed with the Expressionist Analyst Pro 4.0 software (Genedata). A valid value proportion analysis was performed for each group (4 of 5 probe sets had to show a signal) and the resultant groups of probe sets were united. These data were subjected to a number of pairwise comparisons using the Expressionist Analyst Pro 4.0 software (Genedata). Statistical analyses included pairwise comparisons between SAG- or PAC treated samples and vehicle-treated samples. Probe sets were considered to be regulated if they were outside of the ellipsoid region in the Volcano plot applying the following thresholds: Volcano plot: >5x-fold change and P-value <1×10-5 from T-test for 40 nM SAG and PAC; for 2.5 nM SAG and 4 nM PAC >3-fold change and P-value <5×10-3. Venn intersection analyses of significantly regulated genes were performed to identify genes regulated commonly by different treatments using the Expressionist Analyst Pro 4.0 software. Pathway analyses were performed with the GeneGo Metacore (St. Joseph, MI, USA) database and software tools. All Affymetrix cel-file data are available via the ArrayExpress accession number **E-MTAB-377**.

### Cloning of shRNA constructs

The BLOCK-iT RNAi Designer algorithm (Invitrogen, Carlsbad, CA, USA) was used to analyze TP53 mRNA (GenBank accession number, NM_000546.4) and to identify three target sequences for shRNA, i.e. shTP53_1 (sense) 5′-GCATCTTATCCGAGTGGAAGG-3′ and 5′-CCTTCCACTCGGATAAGATGC-3′; shTP53_2 (sense) 5′-GACTCCAGTGGTAATCTAC-3′ and 5′-GTAGATTACCACTGGAGTC-3′; shTP53_3 (sense) 5′-GCGCACAGAGGAAGAGAATCT-3′ and 5′-AGATTCTCTTCCTCTGTGCGC-3′. The target sequences for a non-specific control shRNA (non-targeting shRNA) were shCtrl1 (sense) 5′-TAAGGCTATGAAGAGATAC-3′ and 5′-GTATCTCTTCATAGCCTTA-3′ and shCtrl2 (sense) 5′-TTCTCCGAACGTGTCACGT-3′ and 5′-ACGTGACACGTTCGGAGAA-3′.

Complementary synthetic DNA oligonucleotides were hybridized and inserted into pENTR/U6 vector (Invitrogen). shRNA cassettes were recombined by Gateway cloning into a modified pLenti-6 destination vector (pGT3, Invitrogen) to generate lentiviral shRNA expression constructs shCtrl1, shCtrl2, shTP53_1, shTP53_2, and shTP53_3. All constructs were confirmed by DNA sequencing at the Services in Molecular Biology (SMB), Berlin, Germany.

### Production of lentivirus and transduction of A549 cells

293FT cells were transfected with different pGT3 expression vectors containing TP53 shRNAs or nontarget control shRNA. Lentivirus production was carried out according to the BLOCK-iT U6 RNAi Entry Vector Kit User Manual (Invitrogen). A549 cells were infected with lentiviruses recombinant for shTP53_1, shTP53_2, shTP53_3, or non-target control shRNAs shCtrl1 and shCtrl2, respectively, and selected with hygromycin (100 µg/ml). Individual clones were expanded and tested for TP53- knockdown efficiency by qRT-PCR (data not shown) and immunoblotting.

## Results

### Sagopilone efficaciously inhibits proliferation of NSCLC cell lines in the nanomolar range

**Figure 1:**
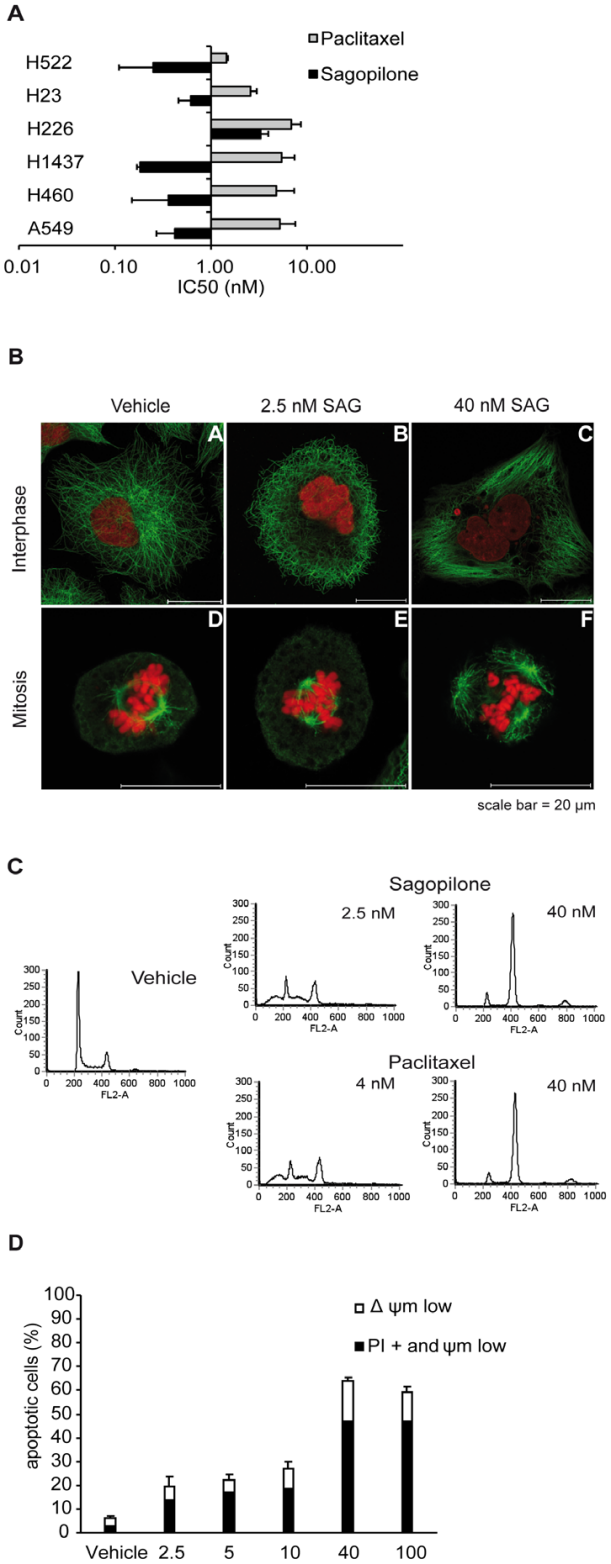
Effect of sagopilone (SAG) on cell proliferation, tubulin cytoskeleton, cell cycle and apoptosis of lung cancer cells. 1A, SAG and PAC inhibit proliferation of lung cancer cell lines. Six different lung cancer cell lines were treated with SAG or PAC for 72 hrs. Proliferation was measured with crystal violet assay. Mean IC50 values as measure of the half-maximal growth inhibition and standard deviations are shown. 1B, Immunofluorescence staining of α-tubulin (green) and DNA (red) in A549 lung cancer cells after incubation with either vehicle (0.1% ethanol), 2.5 nM, or 40 nM SAG. Scale bar = 20 µm. Representative pictures of interphase and mitotic cells are shown. 1C, FACS analysis of A549 cells treated with vehicle, 2.5 and 40 nM SAG or 4 nM and 40 nM PAC for 18 hrs revealed G2/M arrest at 40 nM SAG or PAC and increased numbers of cells with <2N and >2N DNA content and enrichment in G_0_/G_1_ phase of the cell cycle at 2.5 nM SAG or 4 nM PAC. 1D, Induction of apoptosis by increasing concentrations of SAG in A549 cells. A549 cells were incubated for 72 hrs with vehicle, or 2.5 nM, 5 nM, 10 nM, 40 nM, or 100 nM SAG. Further, the cells were stained with 3,3′-dihexyloxacarbocyanine iodide (DiOC6(3)) and propidium iodide for FACS measurement. White bars indicate the mean percentage of cells characterized by decrease of ΔΨm (ΔΨm low) and black bars indicate cells with ΔΨm low and high propidium iodide signal (PI+ and ΔΨm low) due to plasma membrane rupture. The mean of three independent experiments and standard deviation is given.

The anti-proliferative activity of SAG and PAC was examined in 6 non-small cell lung cancer cell lines of different subtypes (adenocarcinoma: A549, NCI-H1437, NCI-H23, NCI-H522; squamous cell carcinoma: NCI-H226; large cell lung carcinoma: NCI-H460) using an *in vitro* proliferation assay (Fig. 1A). SAG inhibited lung tumor cell proliferation in all cell lines, with IC50 values ranging from 0.2 to 3.3 nM, and was effective at sub-nanomolar concentrations (≤1 nM) in five of the six cell lines. Moreover, SAG was consistently more efficacious than PAC.

### Sagopilone interferes with cytoskeletal functions and induces apoptosis in A549 cells

Further experiments on the general mode of action of SAG were performed with the A549 as model cell line. Vehicle-treated A549 cells showed a normal microtubule spread in interphase cells and typical bipolar spindles with congressed chromosomes at the metaphase plate in mitotic cells (Fig. 1B). In contrast, when A549 cells were incubated with 40 nM SAG marked microtubule bundling in interphase cells was visible which led to an abnormal spindle organization in metaphase cells, with multiple spindle poles, several plates of congressed chromosomes, and an irregular chromosomal alignment. These cellular effects were dose-dependent and also seen after incubation with 2.5 nM SAG, but to a lesser extent.

Effects of SAG and PAC on cell cycle progression were measured in A549 cells *in vitro* with FACS analysis (Fig. 1C and Supplementary Fig. S1). Two different phenotypes were observed: Low concentrations of SAG or PAC (0.5–5 nM and 2–7 nM, respectively) induced aberrant cell division resulting in the formation of an increased percentage of aneuploid cells with a DNA content <2N or >2N, but only a slight increase in the percentage of cells in G2/M phase. A different phenotype was observed at higher concentrations of SAG and PAC (>10 nM): Here, a dramatic increase in the percentage of cells in the G2/M phase was observed (Fig. 1C and Supplementary Fig. S1). For all further analyses of the two different phenotypes, two concentrations were employed which induce either the aneuploid phenotype, i.e. 2.5 nM SAG or 4 nM PAC or the mitotic arrest phenotype, i.e. 40 nM SAG or PAC.

A concentration-dependent induction of apoptosis by SAG was observed in FACS analysis after 72 hrs of incubation. Interestingly, treatment with low concentrations of SAG (2.5, 5, and 10 nM) effectively inhibited cell proliferation but induced only little apoptosis, whereas the high concentration SAG treatments (40 nM and 100 nM) led to pronounced induction of apoptosis in A549 cells (Fig. 1D).

### Strong effects of high concentration, but not by low concentration sagopilone-treatment on changes in genome-wide gene expression in A549 cells

**Figure 2:**
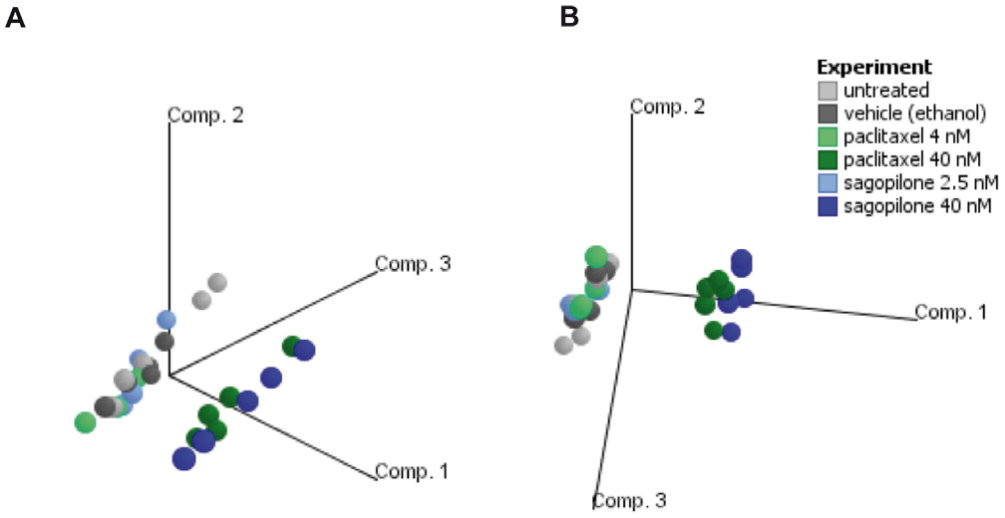
Differential gene expression induced by high versus low concentration of sagopilone. 2A and 2B. Principle Component Analysis (PCA) of microarray data of A549 cells untreated (grey), vehicle-treated (dark grey), or treated with 2.5 nM (blue) or 40 nM SAG (dark blue) and 4 nM (green) or 40 nM PAC (dark green) for 18 hrs. Each plotted sphere represents the expression profile of an individual sample with n = 5 independent biological replicates on the projection of the data on the first three principal components, accounting for most of the variability in the data (labeled axes). Views from two different angles (Fig. 2A, 2B) are shown to visualize the clustering.

RNA was isolated from A549 cells, untreated, vehicle control-treated, as well as treated with two concentrations each of SAG (2.5 nM and 40 nM) and PAC (4 nM and 40 nM) for 18 hrs, and hybridized to Affymetrix HGU133Plus2.0 arrays. High-quality gene expression data were obtained. A principle component analysis (PCA) based on the expression of all genes revealed two main clusters: One cluster contained the untreated, vehicle-treated and low concentration SAG (2.5 nM)- or PAC-treated (4 nM) samples, whereas samples treated with high concentrations (40 nM) of SAG or PAC formed a separate cluster (Fig. 2A, 2B) indicating that treatment with a low drug concentration of SAG or PAC induced only relatively small gene expression changes as compared to the untreated samples, whereas a high drug concentration of SAG or PAC induced stronger gene expression changes.

Paired t-tests comparing each treatment group with the vehicle-treated groups were performed and the results displayed as Volcano plot (Supplementary Fig. S2) depicting the significance as a function of the fold change. Four gene lists were generated with the threshold parameters for P-value and fold change of the high and low concentration treatments of both compounds were as described in Table 1, together with the total number and the number of up- and down-regulated genes. The gene lists obtained from the statistical tests were compared using Venn diagrams. From the total of 503 genes regulated by 40 nM PAC and 593 by 40 nM SAG, 391 genes were both regulated by both treatments, indicating a similar set of genes affected by a high concentration of both TBAs. A Gene Ontology (GO) analysis of the 391 regulated genes with the GeneGo Metacore software revealed a similar occurrence and rank order of biological processes (data not shown) indicating a very similar mechanism of action for both TBAs at high concentration. In contrast, when comparing the 593 genes regulated by 40 nM SAG with the 221 genes affected by treatment with 2.5 nM of the same drug, only 41 genes were commonly regulated by both drug concentrations and a meager nine genes were commonly regulated by 40 nM (total of 503 genes) or 4 nM PAC (total of 158 genes), which indicated that treatment with a high versus a low concentration of either SAG or PAC for 18 hrs regulates a non-overlapping set of genes. When comparing the effects of 2.5 nM SAG (total of 221 genes) with 4 nM PAC (total of 158 genes), 30 genes were regulated by both TBAs, reflecting about one-fifth of all low concentration PAC and one-seventh of all low concentration SAG regulated genes. This suggests an overlapping but also differential effect on gene regulation at low concentration of SAG or PAC. Among the 30 genes commonly regulated by SAG and PAC were the TP53 response genes.

**Table 1:** Number of regulated genes after treatment with sagopilone (SAG) or paclitaxel (PAC).

Treatment	P-value	Fold Change	Regulated Genes	Up-regulated	Down-regulated
4 nM PAC vs. vehicle	≤5×10^−3^	≥3	158	83	75
2.5 nM SAG vs. vehicle	≤5×10^−3^	≥3	221	110	111
40 nM PAC vs. vehicle	≤1×10^−5^	≥5	503	403	100
40 nM SAG vs. vehicle	≤1×10^−5^	≥5	593	455	138

Shown is the number of genes that were either up- or down-regulated obtained from Volcano plots (similar to the one shown in Supplementary Figure 2) using specific parameters for P-value and fold change.

### Genes involved in G2/M phase transition are upregulated by high concentration sagopilone treatment

Differentially regulated genes were subjected to pathway analysis using the GeneGo MetaCore software and database. Genes involved in G2/M phase transition and mitosis, such as Cyclin A, Cyclin B, Nek2A and Securin [23]; [24] and genes such as BUB1, BUBR1 and CDC20, which are components of the spindle assembly checkpoint (SAC) [25], were upregulated after treatment with high concentration of SAG or PAC (Table 2). CDK1, which is essential for the G1/S and G2/M phase transitions of eukaryotic cells, is down-regulated by 40 nM SAG or PAC.

**Table 2:** Differently expressed genes with relevance to G2/M transition or mitosis after treatment with high concentrations of sagopilone (SAG) or paclitaxel (PAC).

Identifier	Gene Name	Gene Symbol	Fold Change 40 nM SAG	Fold Change 40 nM PAC
232588_at	stromal antigen 1	STAG1	12.50	11.37
207331_at	centromere protein F, 350/400 ka (mitosin)	CENPF	5.59	5.25
232466_at	Cullin 4A	CUL4A	4.90	4.66
1556339_a_at	Ubiquitin-activating enzyme E1C (UBA3 homolog, yeast)	UBE1C	4.03	3.89
215623_x_at	SMC4 structural maintenance of chromosomes 4-like 1 (yeast)	SMC4L1	3.97	3.75
244427_at	Kinesin family member 23	KIF23	3.92	3.33
233940_at	Echinoderm microtubule associated protein like 4	EML4	3.51	3.20
242362_at	Cullin 3	CUL3	2.74	n.c.
228729_at	cyclin B1	CCNB1	2.52	2.35
204641_at	NIMA (never in mitosis gene a)-related kinase 2	NEK2	2.25	2.09
221258_s_at	kinesin family member 18A	KIF18A	n.c.	2.09
218755_at	kinesin family member 20A	KIF20A	2.09	1.93
236974_at	Cyclin I	CCNI	2.01	n.c.
209408_at	kinesin family member 2C	KIF2C	1.97	1.84
208079_s_at	serine/threonine kinase 6 (aurora kinase A)	STK6	1.96	1.86
209642_at	BUB1 budding uninhibited by benzimidazoles 1 homolog	BUB1	1.92	1.69
202870_s_at	CDC20 cell division cycle 20 homolog (S. cerevisiae)	CDC20	1.91	1.79
203755_at	BUB1 budding uninhibited by benzimidazoles 1 homolog beta	BUB1B	1.91	1.69
204170_s_at	CDC28 protein kinase regulatory subunit 2	CKS2	1.86	1.81
203418_at	cyclin A2	CCNA2	1.86	1.84
210052_s_at	TPX2, microtubule-associated protein homolog (Xenopus laevis)	TPX2	1.75	1.67
218355_at	kinesin family member 4A	KIF4A	1.71	1.57
202705_at	cyclin B2	CCNB2	1.65	1.64
203554_x_at	pituitary tumor-transforming 1 (Securin)	PTTG1	1.52	1.50
209714_s_at	cyclin-dependent kinase inhibitor 3	CDKN3	1.50	n.c.
223394_at	SERTA domain containing 1	SERTAD1	n.c.	1.35
203967_at	CDC6 cell division cycle 6 homolog (S. cerevisiae)	CDC6	0.48	0.49
213523_at	cyclin E1	CCNE1	0.46	0.44
210559_s_at	cell division cycle 2, G1 to S and G2 to M	CDC2	n.c.	0.46
203213_at	cell division cycle 2, G1 to S and G2 to M	CDC2	0.42	n.c.
202107_s_at	MCM2 minichromosome maintenance deficient 2	MCM2	0.45	0.49
201930_at	MCM6 minichromosome maintenance deficient 6	MCM6	0.44	0.45
205296_at	retinoblastoma-like 1 (p107)	RBL1	0.36	0.40
205034_at	cyclin E2	CCNE2	0.22	0.24

From the overall 705 differentially regulated genes in response to treatment with 40 nM SAG or PAC (assessed by pair wise comparisons of treatment versus vehicle and selection according to Volcano plot with >5fold change, P-value<1×10-5 after Affymetrix gene expression analysis) the 34 genes involved in G2/M transition and mitosis (Gene Ontology classification cell cycle, mitosis or cytokinesis) are shown.

Statistical significance level P<0.001. n.c. =  no change.

**Figure 3:**
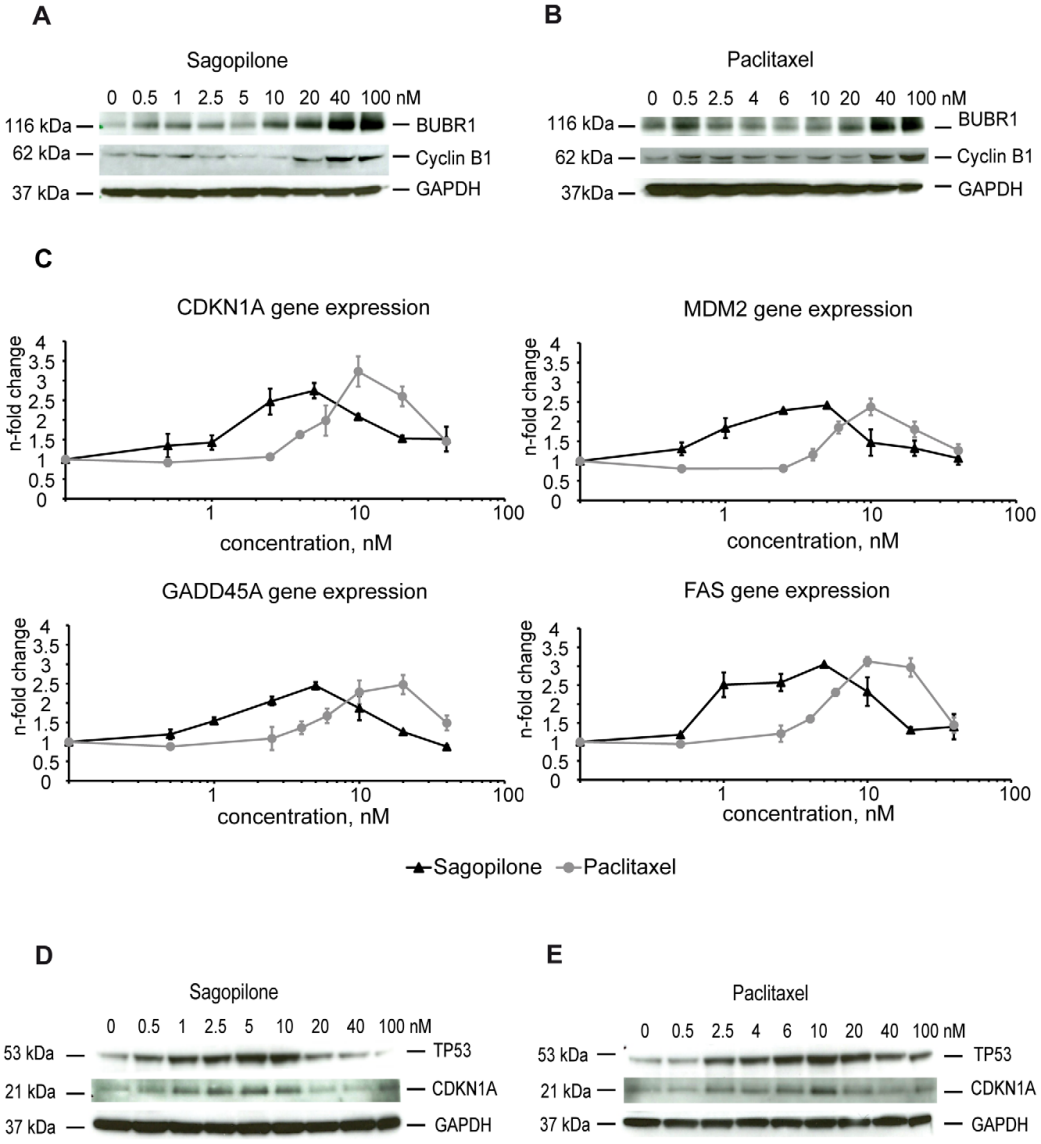
Validation of differential gene regulation by sagopilone and paclitaxel on the RNA and protein level. 3A, 3B, Increase of Cyclin B1 protein and BUBR1 protein by high concentrations of SAG (Fig. 3A) and PAC (Fig. 3B) A549 cells were incubated with the indicated concentrations of SAG or PAC for 18 hours. Cell lysates were subjected to immunoblotting and probed with antibodies recognizing Cyclin B1 and BUBR1, respectively. GAPDH served as loading control. 3C, Regulation of TP53 target genes by SAG and PAC. A549 cells were incubated with the indicated concentrations of SAG or PAC for 18 hours and subjected to RNA extraction. Expression of CDKN1A, MDM2, GADD45A, and FAS, was determined by real-time PCR (TaqMan) and normalized to the expression of the endogenous control gene HPRT. The mean of three independent experiments and standard deviations are shown. The fold change of the vehicle treated A549 cells was set as 1.0 and the SAG treated samples were normalized to the vehicle treated A549 cells. 3D, 3E, Increase of TP53 and CDKN1A protein levels by concentrations of SAG or PAC between 0.5–10 nM. A549 cells were incubated with the indicated concentrations of SAG (Fig 3D) or PAC (Fig. 3E) for 18 hours. Cell lysates were subjected to immunoblotting and probed with antibodies recognizing TP53, CDKN1A and GAPDH, respectively.

As treatment with 40 nM SAG or PAC led to mitotic arrest in A549 cells we were interested in analyzing the protein expression levels of the differentially expressed genes involved in G2/M phase of the cell cycle in dependence of the drug concentration. Both, SAG and PAC, at 40 nM markedly upregulated protein expression of Cyclin B1 and BUBR1 (Fig. 3A and 3B) as well as mRNA expression (Supplementary Fig. S4), whereas treatments at concentration below 10 nM SAG or 20 nM PAC did not significantly alter the expression levels of the proteins (Fig. 3A and 3B) and mRNAs (Supplementary Fig S4). The results revealed that high concentrations of A549 cells with SAG or PAC - concurrent with the observed G2/M arrest - led to an upregulation of genes and proteins involved in the G2/M phase.

### Genes involved in DNA damage response are upregulated by high concentration sagopilone treatment

Interestingly, GO analysis revealed that genes involved in DNA damage response and repair pathways such as the human DNA Polymerase epsilon (POLE) [26], XRCC6 and XRCC5 [27], were found to be upregulated after treatment of A549 cells with 40 nM SAG or PAC (Table 3). In order to analyze a potential direct effect of SAG on DNA damage, phoshorylation of histone H2AX was measured as marker for DNA double strand breaks (DSBs). In A549 cells, high concentrations of SAG, i.e. at 40 nM and 100 nM, increased phosphorylations of H2AX (Supplementary Fig. S3A). Treatment with the pan-caspase inhibitor zVAD-fmk inhibits both phosphorylation of H2AX and PARP cleavage demonstrating that the SAG-induced increase in DSBs is not a direct effect of SAG, but rather a consequence of the increased DSBs which accompanies the apoptosis.

**Table 3:** Differently expressed genes involved in DNA damage response after treatment with high concentrations of sagopilone (SAG) or paclitaxel (PAC).

Identifier	Gene Name	Gene Symbol	Fold Change 40 nM SAG	Fold Change 40 nM PAC
1560509_at	Polymerase (DNA directed), epsilon	POLE	5.88	4.24
237133_at	Sterile alpha motif and leucine zipper containing kinase AZK	ZAK	3.88	4.38
215308_at	Thyroid autoantigen 70 kDa (Ku antigen)	G22P1	3.37	3.16
232633_at	X-ray repair complementing defective repair in Chinese hamster cells 5	XRCC5	3.02	2.82
207746_at	polymerase (DNA directed), theta	POLQ	2.66	2.43
204317_at	G-2 and S-phase expressed 1	GTSE1	2.18	n.c.
211040_x_at	G-2 and S-phase expressed 1	GTSE1	n.c.	1.99
203554_x_at	pituitary tumor-transforming 1	PTTG1	1.52	1.50
208808_s_at	high-mobility group box 2	HMGB2	1.51	n.c.
204767_s_at	flap structure-specific endonuclease 1	FEN1	n.c.	0.59
205698_s_at	mitogen-activated protein kinase kinase 6	MAP2K6	0.48	0.52

From the overall 705 differentially regulated genes in response to treatment with 40 nM SAG or PAC (selected by pairwise comparison and Volcano Plot analysis, see Table 1), genes were further selected using the search phrases “DNA damage”, “double strand breaks”, “DNA repair” and “excision repair” in their Gene Ontology classification. Statistical significance level P<0.01. n.c. =  no change.

### TP53 and direct transcriptional targets of TP53 are upregulated by low concentration sagopilone treatment

Numerous genes that are direct transcriptional targets of TP53 [28], such as CDKN1A, or GADD45A were upregulated after treatment of A549 cells with low concentration of SAG or PAC on the mRNA level (Table 4). Remarkably, A549 cells treated with 2.5 nM SAG showed a more pronounced upregulation of TP53 target genes compared to cells treated with 4 nM PAC (Table 4).

**Table 4:** Differential expression of TP53 target genes after incubation with low concentrations of sagopilone (SAG) and paclitaxel (PAC).

Identifier	Gene Name	Gene Symbol	Fold Change 2.5 nM SAG	Fold Change 4 nM PAC
225912_at	tumor protein p53 inducible nuclear protein 1	TP53INP1	2.82	1.90
217373_x_at	Mdm2, transformed 3T3 cell double minute 2	MDM2	2.47	n.c.
202284_s_at	cyclin-dependent kinase inhibitor 1A (p21, Cip1)	CDKN1A	2.30	1.44
215719_x_at	Fas (TNF receptor superfamily, member 6)	FAS	2.20	1.32
201236_s_at	BTG family, member 2	BTG2	2.18	n.c.
207813_s_at	ferredoxin reductase	FDXR	1.79	1.34
203725_at	growth arrest and DNA-damage-inducible, alpha	GADD45A	1.64	n.c.
227345_at	tumor necrosis factor receptor superfamily, member 10d	TNFRSF10D	1.57	n.c.
219628_at	p53 target zinc finger protein	WIG1	1.51	n.c.
223342_at	ribonucleotide reductase M2 B (TP53 inducible)	RRM2B	1.47	n.c.
208478_s_at	BCL2-associated X protein	BAX	n.c.	1.29
203409_at	damage-specific DNA binding protein 2, 48 kDa	DDB2	1.29	1.30
209295_at	tumor necrosis factor receptor superfamily, member 10b	TNFRSF10B	1.28	n.c.
1563016_at	Acetyl-Coenzyme A carboxylase alpha	ACACA	0.21	0.70

From the overall 349 differentially regulated genes in response to treatment with 2.5 nM SAG or PAC (selected by pairwise comparison and Volcano Plot analysis, see Table 1), genes were further selected according to their transcriptional activation by TP53 (Riley et. al., 2008).

Statistical significance level P<0.05, n.c. =  no change.

In order to analyze the effects of SAG and PAC on TP53 target genes in a concentration-dependent manner, A549 cells were treated with increasing concentration of SAG or PAC which revealed a bell-shaped curve of gene induction for the TP53 target genes CDKN1A, MDM2, GADD45A, and FAS: Expression was induced between 1–10 nM SAG or PAC, whereas concentrations exceeding 10 nM SAG or PAC resulted in a lower induction of gene expression (Fig. 3C). Treatment with PAC was consistently less potent in induction of these four genes.

In order to analyze if the changes observed on the RNA level were also mirrored by changes on the protein level, immunoblot analysis of TP53 and CDKN1A were performed from lysates of A549 cells treated with increasing concentrations of SAG or PAC for 18 hrs. TP53 and CDKN1A showed increased protein expression levels after treatment with 0.5–10 nM SAG or PAC (Fig. 3D and 3E).

### Knockdown of TP53 increases apoptosis induction by low concentration of sagopilone in A549 cells

**Figure 4:**
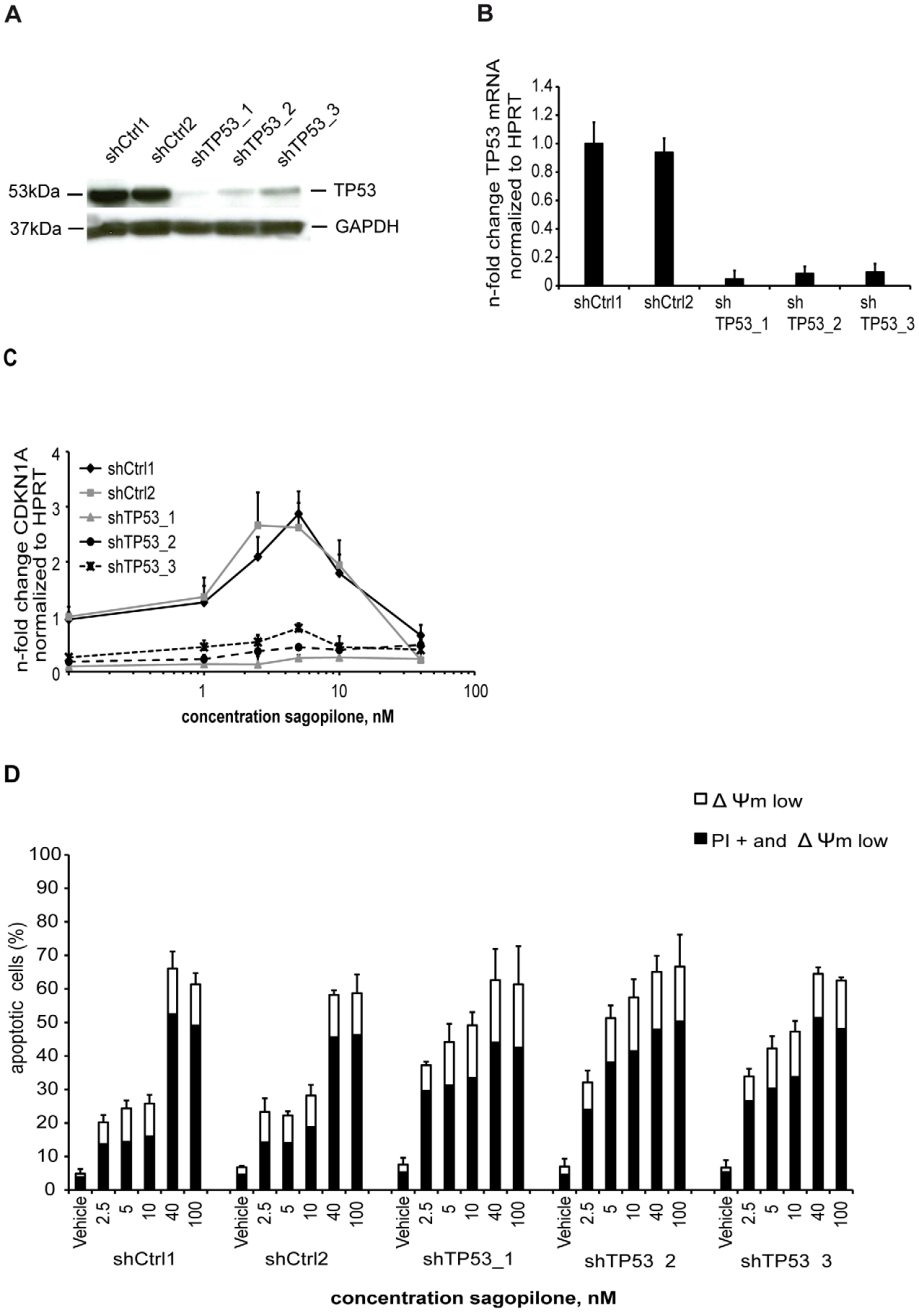
Knockdown of TP53 increases apoptosis induction by low concentration sagopilone. 4A; shRNA mediated knockdown of TP53 in A549 cells after lentiviral transduction and hygromycin selection. A549 cell were stably transduced with three different shRNAs targeting the mRNA of TP53 (shTP53_1, shTP53_2, shTP53_3) or two different control shRNAs (shCtrl1 or shCtrl2). TP53 protein is strongly downregulated in A549 cells stably transfected with shRNAs targetting TP53. Lysates from A549 cells with shRNA-mediated TP53 knockdown and sh control cells were subjected to immunoblotting and probed with antibodies recognizing TP53 and GAPDH, respectively. 4B, TP53 mRNA is downregulated in A549 shTP53 cells. TP53 gene expression in A549 shTP53 knockdown and control cell lines were determined by real-time PCR (TaqMan) and normalized to the endogenous control (HPRT). The mean TP53 expression of shCtrl1 was set as 1.0 and the TP53 expression of the control shRNA or TP53 shRNA was normalized to the expression of shCtrl1. Shown is the average of three independent experiments and standard deviations. 4C, TP53 knockdown inhibits CDN1A induction by SAG. Regulation of TP53 target gene CDKN1A by SAG. A549 shTP53 knockdown and control cell lines cells were incubated with the indicated concentrations of SAG for 18 hours and subjected to RNA extraction. Expression of CDKN1A was determined by real-time PCR (TaqMan), normalized to the endogenous control (HPRT). Shown is the average of three independent experiments and standard deviations. The average expression of the control shRNA shCtrl1 was set as 1.0 and the SAG treated shTP53 knock down cell lines were normalized to the control. 4D. Knockdown of TP53 increases apoptosis induction by low concentration SAG Apoptosis induction by SAG after 72 hours. A549 sh RNA controls (shCtrl1, shCtrl2) and TP53 shRNA knockdown cell lines (shTP53_1, shTP53_2, shTP53_3) were treated for 72 hrs with vehicle, or 2.5 nM, 5 nM, 10 nM, 40 nM, or 100 nM SAG. Afterwards, the cells were stained with DiOC_6_(3) and propidium iodide for FACS detection of apoptosis-associated mitochondrial membrane potential dissipation (Δ Ψm low) and in combination with plasma membrane rupture (PI + and Δ Ψm low). White bars indicate the mean percentage of cells characterized by decrease of ΔΨm (ΔΨm low) and black bars indicate cells with ΔΨm low and high propidium iodine signal (PI+) due to plasma membrane rupture. Three independent experiments were performed. For statistic significance One-way ANOVA analysis followed by the Bonferroni a posteriori test was performed comparing the response of the control A549 cell line with the shTP53 cell lines treated with the same SAG concentration or vehicle. The mean apoptosis induction was significantly higher in the three shTP53 cell lines treated with 5 nM SAG than in the control group treated with 5 nM SAG (ANOVA, F = 56.68, p<0.0002), shTP53_1 vs control (ANOVA/Bonferroni, t = 8.195, p<0.01), shTP53_2 vs control (ANOVA/Bonferroni, t = 11.16, p<0.01), shTP53_3 vs control (ANOVA/Bonferroni, t = 7.405, p<0.01). The mean apoptosis induction was significantly higher in two shTP53 cell lines treated with 10 nM SAG than in the control group treated with 10 nM SAG (ANOVA, F = 18,81, p<0.0032), shTP53_1 vs control (ANOVA/Bonferroni, t = 5.171, p<0.05), shTP53_2 vs control (ANOVA/Bonferroni, t = 6.992, p<0.05), while no significant difference was observed between shTP53_3 vs control (ANOVA/Bonferroni, t = 4.750, p>0.05).

To further elucidate the role of TP53 activation in response to SAG, A549 cells containing wild-type TP53 were stably transfected with expression plasmids containing short hairpin RNAs (shRNAs) targeting the mRNA of TP53 for knockdown. The TP53 protein expression level (Fig. 4A) and the TP53 mRNA (Fig. 4B) were dramatically reduced (80–90%) in the three independently generated shTP53 A549 cell lines (Fig. 4A, 4B). Moreover, the transcriptional induction of CDKN1A by SAG was markedly diminished in the TP53 shRNA knock-down cell lines when compared with the two control A549 cell lines shCtrl1 and shCtrl2, in which where p21 was found to be elevated in the expected manner (Fig. 4C).

The effect of increasing concentrations of SAG on the TP53 knockdown compared to the control shRNA cell lines on apoptosis induction was measured by FACS. The TP53 shRNA knockdown cell lines exhibited a significant increase of apoptotic cell numbers compared to the control A549 when treated with 2.5 nM, 5 nM, or 10 nM SAG (Fig. 4D). Treatment with SAG at 40 nM and 100 nM led only to marginally elevated induction of apoptosis in TP53 shRNA knock-down cell lines compared to control cell lines. The results indicate that activation of TP53 and downstream effectors by low concentrations of SAG is responsible for the apoptosis resistance of A549 cells and might represent a mechanism of resistance to SAG.

## Discussion

Resistance towards chemotherapy is a main obstacle in successful lung cancer treatment. Hence the development of new therapeutic regimens should be accompanied by research on molecular mechanisms of resistance at the earliest time point. This could help to identify patient population most likely benefit from treatment and therefore increasing the chance for a more successful therapeutic response.

In this study, the cellular and molecular mechanisms instigated by the new epothilone SAG have further been elucidated and compared with PAC, a standard TBA, used in combination with carboplatin for the treatment of NSCLC. We analyzed the efficacy of SAG compared to PAC in five different lung cancer cell lines *in vitro* and showed that SAG was consistently more efficient than PAC. This is in line with previous articles reporting that SAG has a higher affinity and selectivity towards the target β-tubulin, which results in a higher intracellular drug concentration of SAG compared to PAC [29].

In the past, TBAs were generally believed to cause mitotic arrest, but more detailed studies have identified two different, concentration-dependent phenotypes [30]. We were able to show that this is also the mode of action of SAG in lung cancer cells, where an aneuploid phenotype is induced by 2.5 nM SAG or 4 nM PAC and, in contrast, a mitotic arrest phenotype is induced by 40 nM SAG or PAC, indicating that SAG has a similar concentration-dependent mechanism as PAC. However, we showed that for PAC the induction of aneuploid cells peaked at higher concentrations compared to SAG, probably reflecting the pharmacological differences reported earlier [29].

To explore the differences between the two phenotypes caused by SAG, we have generated genome-wide gene expression profiles of A549 cells treated with low and high concentrations of SAG or PAC, which were analyzed statistically, as well as by pathway analysis tools including Gene Ontology. Treatment of A549 cells with 40 nM SAG or PAC for 18 hrs strongly induced differential gene expression and very similar gene expression profiles by both, SAG and PAC. Due to the fact that the majority of the cells arrested at the metaphase/anaphase transition after treatment, the gene expression patterns mainly showed upregulation of components of the SAC and genes involved in mitosis, like BUBR1 and Cyclin B1, all indicative of a mitotic arrest phenotype induced by 40 nM SAG or PAC. These results are in line with previous reports about gene expression studies comparing epothilones and PAC [31]; [32]. Moreover, a recent report about primary NSCLC mouse xenograft models treated with SAG unveiled a highly significant upregulation of genes involved in pathways like SAC and chromosome segregation in the primary NSCLC xenograft models which are SAG responders compared to non-responder [7], indicating that the phenotype observed *in vitro* after treatment with the high concentration of SAG is mainly responsible for tumor cells killing.

Induction of DNA damage response genes and the phosphorylation of histone H2AX, which marks DNA as prerequisite for repair process to take place, at 40 nM SAG, as reported here, might be due to direct induction of DNA damage by high concentration of SAG. As treatment with the pan-caspase inhibitor zVAD-fmk inhibits both phosphorylation of H2AX and PARP cleavage indicates that the SAG-induced increase in DSBs is not direct effect of SAG, but rather the consequence of the increased apoptosis. However, up to now, the role of DSBs and the phosphorylation of H2AX in response to SAG and its potential role in the mechanistic activity of SAG will need further investigation.

In our studies, treatment of A549 cells with low concentrations of SAG or PAC resulted in stabilization of TP53 and induction of TP53 target genes, potentially resulting from consistent translation of the long-lived TP53 mRNA during prolonged mitosis induced by both drugs [33]; [34]; [35]. However, it should be noted that induction of TP53 target genes was more pronounced after SAG treatment.

This upregulation of TP53 target genes such as CDKN1A or GADD45A, mostly resembled an activation pattern which is caused in response to mild, repairable damage, and induced cell cycle arrest, rather than strong damages which promote apoptosis [36]; [28]; [37]. This allows repair processes to take place and the cells to survive. In terms of chemotherapy this would indicate an unfavorable condition, because the cells might start regrowing after a terminal cell cycle arrest. The aneuploid cells finally arrest in the G1 state due to a postmitotic checkpoint that is dependent on TP53 [38]. TP53 mediates G1 arrest mainly by increasing protein levels of the cyclin-dependent kinase (CDK) inhibitor p21 (CDKN1A) [39]. Apart from functions in cell cycle regulations several anti-apoptotic functions of p21 have been described [40]; [41]; [42]; [43]. Moreover, the weak apoptosis induction after low concentration SAG treatment of A549 cells compared to high concentrations leads to the conclusion that anti-apoptotic effects of TP53 overweigh in this phenotype.

To date the role of TP53 in the sensitivity of cancer cells to TBAs is contested [14]; [15]. Many groups reported that cells lacking wild type TP53 displayed increased sensitivity to PAC [16]; [44]; [45]. Sensitization of TP53 wild type (wt) cells to low concentration PAC was achieved by by siRNA-mediated knock-down of TP53 in NCI-H460 cells [46]. Furthermore, the gene transfection of TP53-null human non-small cell lung cancer H358 cells with wt TP53 resulted in loss of PAC sensitivity [47].

To address the question whether TP53 plays a role in the sensitivity towards SAG, we analyzed the effect of TP53 knock down on the apoptosis induction of A549 cells. We have shown that the knockdown of TP53 increased the rate of apoptosis after low concentration SAG treatment in A549 cells. These effects in A549 TP53 knockdown cells were mostly based on abrogation of TP53-mediated transcription at low concentration of SAG. The TP53-dependent CDKN1A induction was coincident with resistance to low concentration SAG-induced apoptosis in A549 cells. Thus, the transactivation of TP53 is responsible for the low apoptosis induction of A549 cells *in vitro* after treatment with low concentration SAG. Pharmacological inhibition of TP53 using pifithrin-α could be a second method to validate the role of TP53 in SAG efficacy. Results from previous studies show that the sensitizing effects of pifithrin-α towards microtubule inhibiting drugs [48]; [46] is well in accordance with the findings of our study for SAG in NSCLC.

On the other hand it has been shown that pifithrin-α is not completely specific in its action on TP53, as it is known to have targets other than TP53 [49]; [50]; [51] and pifithrin-α is known to protect cells from DNA damage-induced apoptosis by a p53-independent mechanism [52]. Therefore in the current study we used shRNA based knockdown of TP53 as the most specific method on otherwise genetically identical cell lines.

It might be possible that in tumors, harboring areas with low vascularization only very low amounts of SAG will actually reach the tumor cells. In that case, the aneuploid phenotype (here experimentally induced by 2.5 nM SAG or 4 nM PAC) would subsequently result in a G1 arrest. Under that condition, the TP53 response would play an important role. It is an open question whether *in vivo* these cells then die from apoptosis or were arrested for a certain time and start regrowing eventually, which is well in accordance with data from a recent study in patient derived NSCLC xenografts showing a better long-term response to SAG in models with mutated TP53 [7].

About half of all NSCLC cases harbor mutations in TP53 [13]. The question remains whether these tumors might have a higher probability to respond to SAG. SAG is currently in clinical development and has been evaluated in phase II trials in NSCLC [53]; [54] therefore investigations whether mutational status of TP53 could serve as predictive biomarker in clinical trials warrants further investigation. Additionally, it could be of clinical relevance if patients with TP53 wild type tumors benefit from combination therapy with drugs inhibiting TP53. Those drugs would enhance the effect of SAG therapy and concurrently would help to reduce the systemic chemotherapy-induced toxicity [55]. As the currently available TP53 inhibitors such as pifithrin-α are not appropriate for clinical application. TP53 inhibitors that more specifically inhibit certain functions of TP53 i.e. those that block TP53-dependent transactivation (with no effect on p53-mediated apoptosis) are needed.

## Supporting Information

Figure S1 **Cell cycle analysis of A549 cells treated with different concentrations sagopilone and paclitaxel.** Cells were incubated with growth medium containing 0 to 100 nM SAG and PAC for 18 hours, followed by fixation and incubation with propidium iodide. DNA content was determined by flow cytometry. The amounts of cells constituting the aneuploid, G1, S and G2/M populations were determined using ModFit software and plotted against the drug concentration. S phase cells are not shown because of clarity. Mean values and standard deviation given.
(TIF)

Figure S2 **Volcano plot from T-test of 40 nM sagopilone vs. vehicle (threshold: >5-fold change, P-value <1×10^−5^).** The Volcano plot depicts the significance as a function of the fold change. Thus, highly significant genes with a low fold change as well as genes which possess a high fold change and a relatively low significance were indicated in red. Thresholds for the Volcano plots were defined as ellipse with >5-fold change and P-value <1×10^−5^ from T-test for 40 nM SAG and PAC and for 2.5 nM SAG and 4 nM PAC as ellipse with >3-fold change and P-value <5×10^−3^.
(TIF)

Figure S3 **γH2AX as marker for DNA double strand breaks and apoptosis.** (A) A549 cell were treated with increasing concentrations of SAG for 18 hours and subjected to western blot analysis. γH2AX antibody staining is shown. (B) Western blot analysis of A549 cells treated with 40 nM SAG for different times in the presence (+zVAD) (40 µM) or absence (-zVAD) of ZVAD.fmk and probed with antibodies detecting PARP, γH2AX, MPM2, respectively. GAPDH served as loading control.
(TIF)

Figure S4 **Regulation of gene expression of Cyclin B1, BUBR1 and TP53 by sagopilone and paclitaxel.** A549 cells were incubated continuously with medium containing increasing concentrations of both agents for 18 hours and were subjected to RNA extraction. After performing a reverse transcription the cDNA was subjected to real time PCR (TaqMan) of Cyclin B1, BUBR1 and TP53. Shown is the fold change compared to the vehicle treated samples as mean of three independent experiments and standard deviations.
(TIF)
