# Supplementary figures and images for: Molecular Mode of Action and Role of TP53 in the Sensitivity to the Novel Epothilone Sagopilone (ZK-EPO) in A549 Non-Small Cell Lung Cancer Cells
Source: PLoS One. 2011 Apr 29;6(4):e19273. doi: 10.1371/journal.pone.0019273 (PMC3084814; doi:10.1371/journal.pone.0019273)

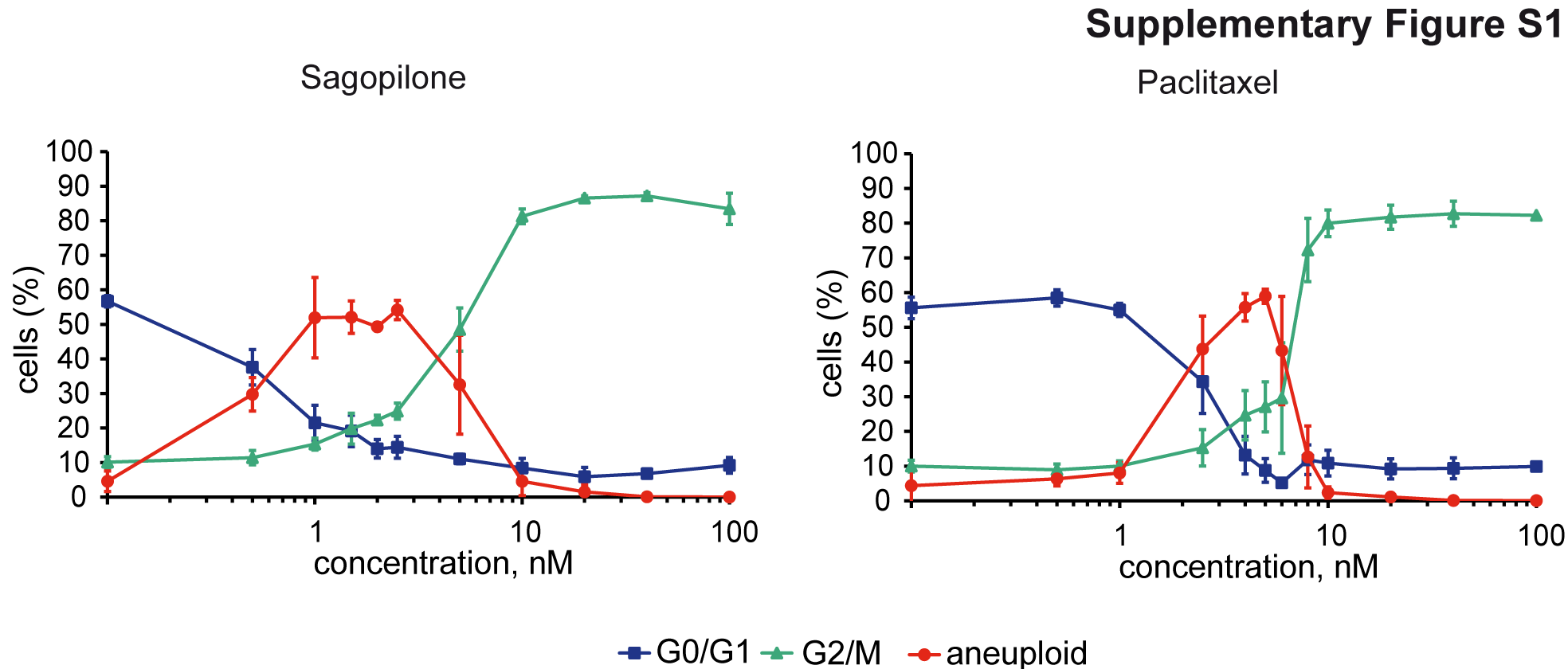

Supplement: Figure S1 — Cell cycle analysis of A549 cells treated with different concentrations sagopilone and paclitaxel. Cells were incubated with growth medium containing 0 to 100 nM SAG and PAC for 18 hours, followed by fixation and incubation with propidium iodide. DNA content was determined by flow cytometry. The amounts of cells constituting the aneuploid, G1, S and G2/M populations were determined using ModFit software and plotted against the drug concentration. S phase cells are not shown because of clarity. Mean values and standard deviation given. (TIF) [file pone.0019273.s001.tif]

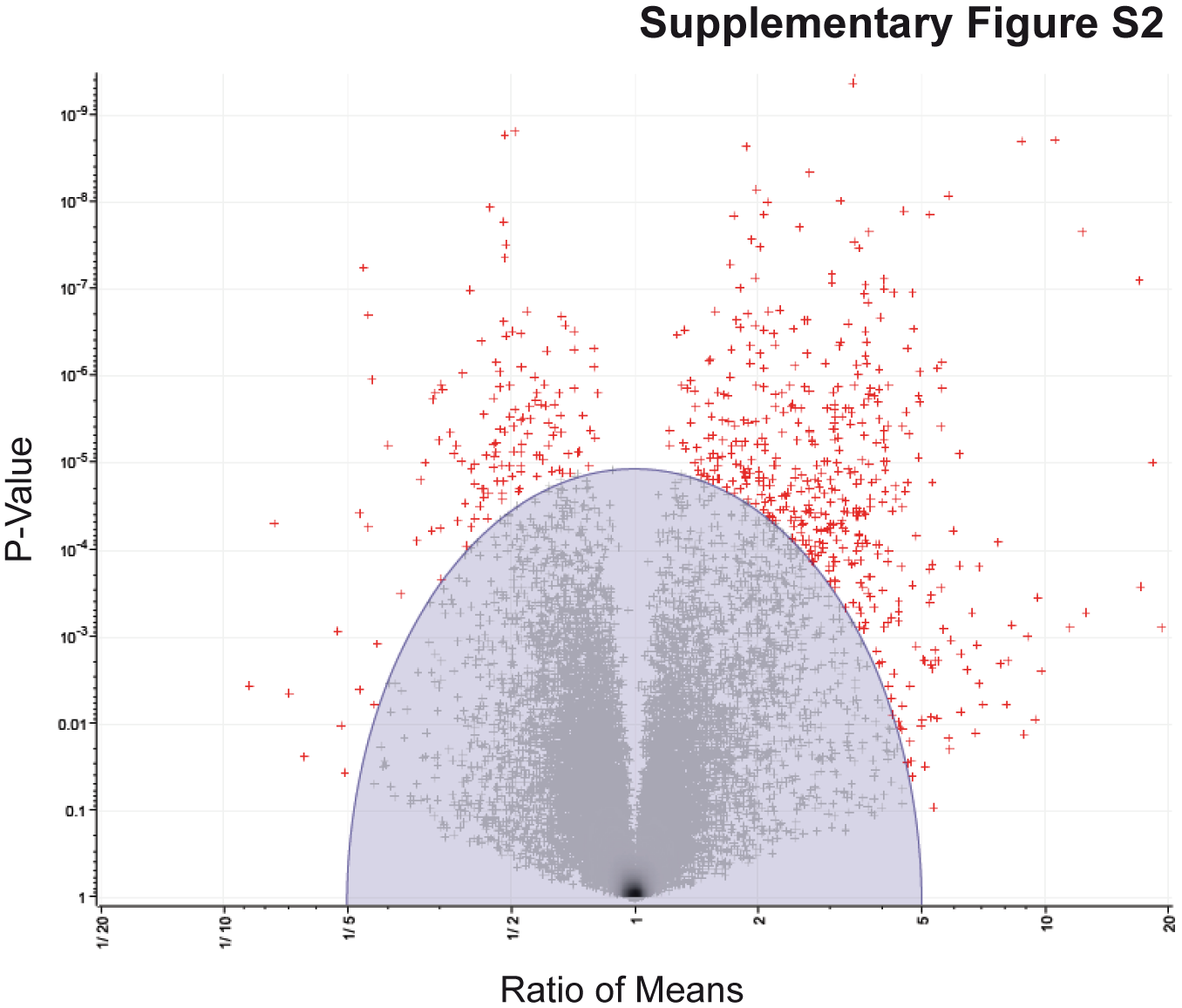

Supplement: Figure S2 — Volcano plot from T-test of 40 nM sagopilone vs. vehicle (threshold: >5-fold change, P-value <1×10^−5^). The Volcano plot depicts the significance as a function of the fold change. Thus, highly significant genes with a low fold change as well as genes which possess a high fold change and a relatively low significance were indicated in red. Thresholds for the Volcano plots were defined as ellipse with >5-fold change and P-value <1×10^−5^ from T-test for 40 nM SAG and PAC and for 2.5 nM SAG and 4 nM PAC as ellipse with >3-fold change and P-value <5×10^−3^. (TIF) [file pone.0019273.s002.tif]

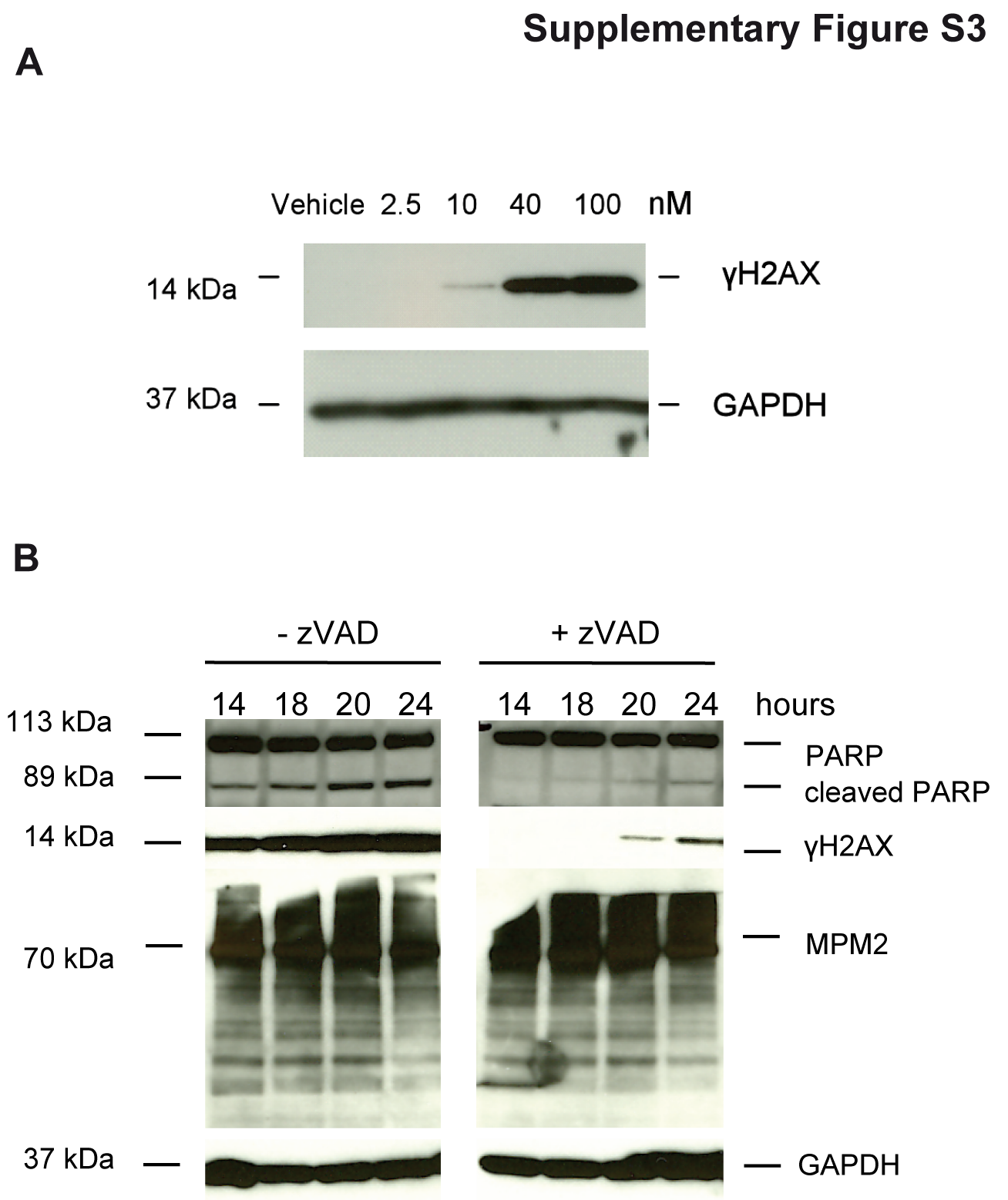

Supplement: Figure S3 — γH2AX as marker for DNA double strand breaks and apoptosis. (A) A549 cell were treated with increasing concentrations of SAG for 18 hours and subjected to western blot analysis. γH2AX antibody staining is shown. (B) Western blot analysis of A549 cells treated with 40 nM SAG for different times in the presence (+zVAD) (40 µM) or absence (-zVAD) of ZVAD.fmk and probed with antibodies detecting PARP, γH2AX, MPM2, respectively. GAPDH served as loading control. (TIF) [file pone.0019273.s003.tif]

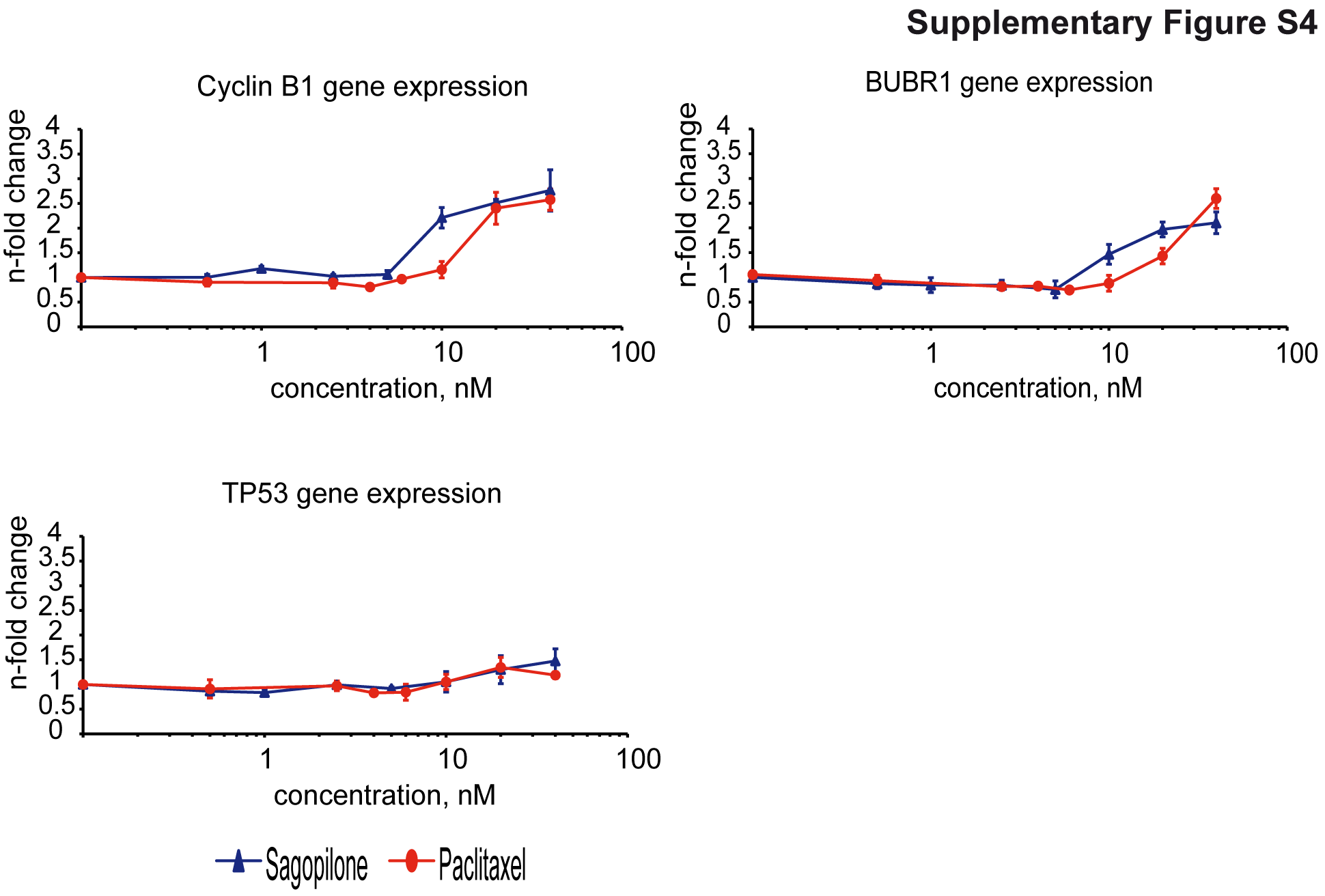

Supplement: Figure S4 — Regulation of gene expression of Cyclin B1, BUBR1 and TP53 by sagopilone and paclitaxel. A549 cells were incubated continuously with medium containing increasing concentrations of both agents for 18 hours and were subjected to RNA extraction. After performing a reverse transcription the cDNA was subjected to real time PCR (TaqMan) of Cyclin B1, BUBR1 and TP53. Shown is the fold change compared to the vehicle treated samples as mean of three independent experiments and standard deviations. (TIF) [file pone.0019273.s004.tif]
